# Association between advanced lung cancer inflammation index and atherosclerosis cardiovascular disease in U.S. adults: a population-based investigation

**DOI:** 10.3389/fnut.2025.1503844

**Published:** 2025-06-04

**Authors:** Zhongtian Xiang, Sheng Lin, Bei Yang, Siman Zhu, Qian Song

**Affiliations:** ^1^Lung Cancer Center, The Second Affiliated Hospital of Nanchang University, Nanchang, China; ^2^Medical College, China Three Gorges University, Yichang, China; ^3^Department of Critical Care Medicine, Renmin Hospital of Wuhan University, Wuhan, Hubei, China; ^4^School of Basic Medical Sciences, Nanchang Health Vocational and Technical College, Nanchang, China

**Keywords:** NHANES, advanced lung cancer inflammation index, cross-sectional study, atherosclerotic cardiovascular disease, cross-sectional

## Abstract

The advanced lung cancer inflammation index (ALI) is a multifaceted tool that combines indicators of inflammation and nutritional status to evaluate patient’s health conditions. Globally, atherosclerotic cardiovascular disease (ASCVD) remains a leading cause of both morbidity and mortality. Considering the pivotal role of inflammation in cardiovascular disease, investigating the relationship between ALI and ASCVD makes sense. A vast amount of information was gathered from the 1999 to 2018 National Health and Nutrition Examination Survey (NHANES). Univariate and multivariate logistic regression models were employed, supplemented by subgroup analyses and interaction tests. Furthermore, smooth curve fitting was applied to examine linear relationships, while the binary linear regression model was utilized to ascertain the threshold effects. The predictive capabilities of the Systemic Immune-Inflammation Index, Platelet-to-lymphocyte Ratio and ALI for ASCVD were evaluated using Receiver Operating Characteristic (ROC) curves with the area under the curve (AUC) values. A cross-sectional study of 48,845 individuals was included in the study. The ALI demonstrated an inverse association with the likelihood of ASCVD in participants [Odds ratios: 0.87, 95% confidence interval (CI): 0.82 to 0.92]. Sex had a significant interaction impact, according to subgroup analyses (*P* for interaction < 0.05). Furthermore, ALI emerged as a more accurate predictor of ASCVD, with the AUC values of 0.608, surpassing both the systemic immune-inflammation index (AUC = 0.502) and the platelet–lymphocyte ratio (PLR) (AUC = 0.569). This study provides evidence that higher ALI levels are associated with a lower probability of ASCVD prevalence in adult Americans. These findings suggest that ALI may serve as a valuable anthropometric marker for predicting ASCVD prevalence.

## 1 Introduction

Globally, atherosclerotic cardiovascular disease (ASCVD) is still a leading cause of morbidity as well as death, placing a substantial strain on healthcare systems and societies (1). The guidelines from the American College of Cardiology and the American Heart Association state that the diagnostic criteria for ASCVD include the occurrence of at least one of the following conditions: angina, myocardial infarction, cerebrovascular accident, or coronary heart disease (2). As a chronic inflammatory disorder marked by the buildup of lipids and fibrous tissue within large arteries, ASCVD leads to a range of life-threatening events, such as myocardial infarction, stroke, and peripheral artery disease. Early and accurate diagnosis of ASCVD is crucial for timely intervention and management, which can significantly reduce the incidence of adverse cardiovascular events and improve patient outcomes. Pharmacological modulation of low-density lipoprotein cholesterol levels via statins and proprotein convertase subtilisin/kexin type 9 (PCSK9) inhibitors constitutes a key therapeutic strategy for ASCVD risk reduction (3). In addition, more precise and accurate approaches that address diabetes heterogeneity in people at risk of ASCVD are needed to understand the responses to specific prevention and remission strategies in patient subgroups (4).

Traditional diagnostic methods for ASCVD, such as lipid profiling and imaging techniques, have been the cornerstone of cardiovascular risk assessment. However, these methods often fall short in providing a comprehensive picture of the inflammatory processes underlying atherosclerosis. Laboratory examinations, including the platelet–lymphocyte ratio (PLR) and systemic immune inflammation index (SII), has been utilized more and more widely to evaluate systemic inflammation as well as its contribution to ASCVD. These biomarkers provide important insights into an individual’s inflammatory status and have been associated with cardiovascular risk (5, 6). However, they also present limitations, such as variability in measurements, lack of specificity to atherosclerosis, and potential confounding by other inflammatory conditions (7).

In recent years, advanced lung cancer inflammation index (ALI) has gained recognition as a new biomarker, combining neutrophil-to-lymphocyte ratio (NLR), serum albumin levels, as well as the body mass index (BMI) to evaluate the inflammatory as well as nutritional status of individuals with advanced lung cancer. Given the shared inflammatory pathways between cancer and atherosclerosis, there is growing interest in exploring the potential association between ALI and ASCVD (8, 9). The integration of ALI into cardiovascular diagnostics could offer a more comprehensive approach to understanding the inflammatory mechanisms driving atherosclerosis and potentially improve the accuracy of ASCVD developing prediction (10, 11).

The ALI reflects systemic inflammation by combining BMI, albumin levels, and the NLR. Inflammation plays a critical role in the development of ASCVD by promoting endothelial damage, plaque formation, and vascular stiffness. BMI reflects chronic inflammation caused by obesity, while decreased albumin levels are commonly associated with systemic inflammation and malnutrition, both of which are closely linked to cardiovascular health. Compared to traditional indices like the PLR and the SII, ALI offers a more comprehensive assessment by integrating multiple inflammation-related markers, providing higher sensitivity and specificity in predicting cardiovascular disease risk. While PLR and SII are associated with cardiovascular risk, they focus solely on single blood components or ratios, failing to comprehensively reflect metabolic status and systemic inflammation. As a result, ALI has certain advantages in predicting cardiovascular health.

The association between ALI and cardiovascular outcomes has been demonstrated in various patient populations. For instance, elevated ALI values have been linked to reduced cardiovascular mortality in hypertensive individuals and those receiving percutaneous coronary intervention for the management of acute coronary syndrome (12). Similarly, in individuals with rheumatoid arthritis, ALI was associated with decreased probability of cardiovascular mortality, highlighting its possibility as a prognostic indicator in inflammatory and autoimmune conditions (13). Furthermore, the SII, another biomarker related to ALI, has been identified as a predictor of cardiovascular risk as well as mortality in rheumatoid arthritis with prediabetes and diabetes (14).

The objective of our research is to explore the association between the ALI and ASCVD. By addressing the gap, this study strives to make meaningful contributions to the current body of research on the investigation of it and propose a novel method that could enhance the early detection and management of atherosclerosis, ultimately guiding more effective and personalized treatment strategies (15, 16).

## 2 Materials and methods

### 2.1 Study design and data source

Data from the National Health and Nutrition Examination Survey (NHANES) were used in the investigation. The United States National Center for Health Statistics (NCHS) is in charge of conducting this survey. The NHANES is a thorough study that evaluates the nutritional and overall health of adults and children in the USA. Utilizing a multistage, stratified probability-cluster sampling approach, NHANES guarantees a representative sample of the civilian non-institutionalized population. This method increases the reliability of the survey by selecting participants from various regions, demographics, and socio-economic backgrounds, giving it a broad national scope. These interviews include questions on health, dietary, socioeconomic status, and demographics. Participants undergo health checkup, laboratory tests, as well as further health evaluations. The data collection began in 1999 and occurs in biennial cycles, providing continuous updates on the United States population’s health status. NHANES ensures transparency and accessibility by making its data, survey designs, and methodologies publicly available (17). This allows researchers from various fields to conduct independent analyses and apply the data to different health-related studies. The ethical aspects of the survey are overseen by the NCHS Ethics Review Committee, which provides ethical clearance for the entire process. Participants give written informed consent before taking part in the survey, ensuring compliance with ethical standards in data collection and research. Secondary analyses of NHANES data do not require additional Institutional Review Board (IRB) approval. This is due to the survey’s established ethical clearance, simplifying the use of the data for various secondary research purposes. This ensures efficient reusability of the data while maintaining ethical integrity (18).

The analysis encompassed participants from NHANES cycles spanning the years 1999 to 2018. Only individuals who provided comprehensive information on laboratory results, demographic characteristics, and responses to questionnaires related to ASCVD diagnoses were included in the study. Participants below the age of 20 were excluded from the analysis.

Demographic information, including age, gender, race, poverty status, and smoking habits, was collected by trained interviewers during in-home interviews, using a computer-based personal interview system. Data pertaining to BMI and blood pressure were obtained from physical examinations conducted at the MECs. Insurance status was assessed through questionnaires, with participants who reported having Medicare coverage being asked to present the Medicare cards for verification. Laboratory data were collected to measure levels of albumin and counts of neutrophils and lymphocytes. The random forest imputation method is particularly effective for handling large datasets, providing accurate and reliable imputation results. This method works by training multiple decision trees and predicting missing values based on the observed features of the data, allowing for the effective imputation of missing values while preserving the complex relationships within the data. In this study, we utilized the random forest algorithm to impute missing data, ensuring that the imputation process did not introduce significant bias. Additionally, the imputation procedure demonstrated high reproducibility and transparency. Therefore, due to partial missing data in some variables within this cross-sectional study, random forest imputation were performed in this study.

## 2.2 Advanced lung cancer inflammation index

The ALI is a composite measure designed to assess the combined effects of inflammation, nutritional status, as well as body weight on the risk of ASCVD. It is calculated using the formula: BMI (kg/m^2^) × albumin level (g/dL)/NLR, where the ratio of neutrophil to lymphocyte counts is known as NLR. To adjust for right skewness in the data, ALI values are transformed using the natural logarithm (Ln-transformation). For stratification, we used the conventional quartile method, which involves ranking all participants’ ALI values in ascending order and dividing them into four groups, each containing approximately the same number of participants. This approach ensures the robustness of the results by systematically comparing the impact of different ALI levels on cardiovascular disease risk. It also allows for clearer identification of potential risk factors in the data analysis, enabling an accurate reflection of the underlying relationship between ALI and ASCVD. So participants are categorized into four groups using quartiles: Q1 (≤ 43.96), Q2 (> 43.96 and ≤ 61.46), Q3 (> 61.46 and ≤ 84.39), and Q4 (84.39). This stratification allows for the analysis of ASCVD risk in relation to varying levels of inflammation, nutrition, and body weight.

Considering that ALI in ASCVD demonstrated a skewed distribution characterized by a significant tail, leading to asymmetry in the data, and the presence of extreme values (outliers) with the potential to disproportionately affect the analysis, we implemented a nature logarithmic transformation. This technique effectively reduces the impact of outliers, thereby ensuring more robust and reliable statistical inferences.

### 2.3 Primary outcome

The outcome is the diagnosis of ASCVD, which is based on the 2013 American College of Cardiology Foundation/American Heart Association Guideline, is defined as having at least one of the following diagnoses: coronary heart disease, angina, heart attack, or stroke (Supplementary Table 1A) (2).

### 2.4 Covariate assessment

Covariates, including demographic data (age, sex, race, as well as ratio of household income to poverty), examination, laboratory, as well as questionnaire data (BMI, albumin, neutrophil counts, lymphocyte counts), and diseases (hypertension, hyperlipidemia, and diabetes), were obtained from the NHANES website, with diagnosis criteria for these diseases detailed in Supplementary Table 1A.

### 2.5 Statistical analysis

The study utilized R software (version 4.2) as well as EmpowerStats (edition 4.1) for demographic analysis with Chi-square tests for categorical variables as well as *t*-tests for continuous variables, association analysis with multivariate logistic regression for ALI and ASCVD, non-linear relationship assessment through a weighted generalized additive model with smooth curve fitting (Since ASCVD is a binary variable, smooth curve fitting uses the gam() function from the mgcv package, with the curve fitting term defined using the s() function. If no degrees of freedom are specified, the model automatically determines the degrees of freedom based on the minimum GCV method. The mgcv package creates thin plate regression splines, and by default, the maximum degrees of freedom are set to 10; the penalty is maximized by using generalized cross-validation by default (19)), mediation analysis using a parallel mediation model, stratified analyses to evaluate subgroup associations, with statistical significance set at a two-sided *P*-value < 0.05, and predictive accuracy determined by receiver operating characteristic (ROC) curves calculating area under the curve (AUC) for SII, PLR, and ALI in predicting ASCVD.

## 3 Results

### 3.1 Participant characteristics

**FIGURE 1:**
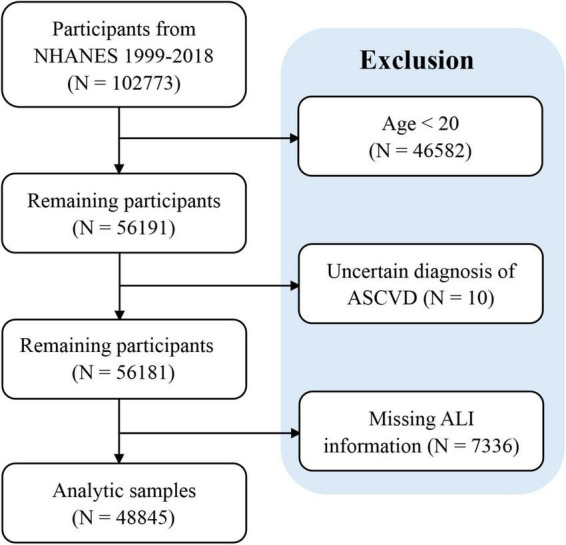
Flowchart of participants inclusion and exclusion. ASCVD, atherosclerotic cardiovascular disease.

From the NHANES 1999 to 2018 data, a total of 48,845 adults were enrolled in our study in accordance with the established inclusion and exclusion criteria. The flowchart of participants inclusion and exclusion was presented in Figure 1. The individuals were divided into 4 quartiles based on Ln-transformed ALI, namely ≤ 43.96 (Q1 group), 43.96–61.46 (Q2 group), 61.46–84.39 (Q3 group) and > 84.39 (Q4 group). Our study included 23562 male and 25283 female individuals. And the overall prevalence of ASCVD was 9.23%, which was observed to weaken with increasing ALI quartiles (Q1 group: 13.48%; Q2 group: 9.88%; Q3 group: 8.58%; Q4 group: 7.77%, *P* < 0.05). Significant differences were identified across ALI quartiles with respect to age, gender, race, smoking status, insurance, hypertension, diabetes, hyperlipidemia, stroke, heart attack, angina, coronary heart disease (all *P* < 0.05) (Table 1).

**TABLE 1:** Baseline demographic and medical characteristics of participants in the NHANES 1999–2018 data.

Characteristics	ALI				*P*-value
	**Q1 group**	**Q2 group**	**Q3 group**	**Q4 group**	
	**[2.83,43.96]**	**(43.96,61.46]**	**(61.46,84.39]**	**(84.39,2842.4]**	
Participants, *N*	12212	12211	12211	12211	
ASCVD, *N* (%)	1644 (13.48%)	1206 (9.88%)	1048 (8.58%)	949 (7.77%)	<0.05
ALI	32.32 ± 8.42	52.71 ± 5.01	71.83 ± 6.52	119.89 ± 71.03	<0.05
Age (years)	52.27 ± 20.16	49.44 ± 18.05	48.33 ± 17.15	47.87 ± 16.44	<0.05
BMI, kg/m2	25.90 ± 5.22	27.97 ± 5.78	29.75 ± 6.54	32.17 ± 7.61	<0.05
Albumin, g/dL	4.14 ± 0.43	4.26 ± 0.34	4.28 ± 0.33	4.26 ± 0.33	<0.05
Neutrophil, k/μL	5.60 ± 2.17	4.49 ± 1.39	3.96 ± 1.28	3.20 ± 1.22	<0.05
Lymphocyte, k/μL	1.64 ± 0.51	2.00 ± 0.54	2.25 ± 0.60	2.75 ± 3.90	<0.05
NLR	3.59 ± 1.55	2.26 ± 0.46	1.77 ± 0.37	1.23 ± 0.35	<0.05
Gender, *N* (%)					<0.05
Male	5763 (47.23%)	5847 (47.88%)	6102 (49.94%)	5850 (47.90%)	
Female	6440 (52.77%)	6364 (52.12%)	6116 (50.06%)	6363 (52.10%)	
Race, *N* (%)					<0.05
Mexican-American	1886 (15.46%)	2313 (18.94%)	2450 (20.05%)	2124 (17.39%)	
Non-Hispanic Black	6784 (55.59%)	5923 (48.51%)	5221 (42.73%)	3855 (31.56%)	
Non-Hispanic White	1537 (12.60%)	1723 (14.11%)	2255 (18.46%)	4306 (35.26%)	
Other	1996 (16.36%)	2252 (18.44%)	2292 (18.76%)	1928 (15.79%)	
Poverty income ratio, *N* (%)					0.101
≤1	2301 (18.86%)	2257 (18.48%)	2232 (18.27%)	2373 (19.43%)	
>1	9902 (81.14%)	9954 (81.52%)	9986 (81.73%)	9840 (80.57%)	
Insurance, *N* (%)					<0.05
Any insurance	10678 (87.50%)	10240 (83.86%)	10178 (83.30%)	10205 (83.56%)	
No insurance	1525 (12.50%)	1971 (16.14%)	2040 (16.70%)	2008 (16.44%)	
Smoking status, *N* (%)					<0.05
Current smoker	2805 (22.99%)	2642 (21.64%)	2411 (19.73%)	2294 (18.78%)	
Former smoker	3279 (26.87%)	2987 (24.46%)	2975 (24.35%)	2918 (23.89%)	
Never smoker	6119 (50.14%)	6582 (53.90%)	6832 (55.92%)	7001 (57.32%)	
Diabetes, *N* (%)					<0.05
Yes	2071 (16.97%)	1920 (15.72%)	2155 (17.64%)	2315 (18.96%)	
No	10132 (83.03%)	10291 (84.28%)	10063 (82.36%)	9898 (81.04%)	
Hyperlipidemia, *N* (%)					<0.05
Yes	7502 (61.50%)	7585 (62.14%)	7807 (63.91%)	7930 (64.94%)	
No	4697 (38.50%)	4621 (37.86%)	4408 (36.09%)	4281 (35.06%)	
Hypertension, *N* (%)					<0.05
Yes	4947 (40.56%)	4710 (38.58%)	4893 (40.06%)	5379 (44.05%)	
No	7250 (59.44%)	7499 (61.42%)	7322 (59.94%)	6832 (55.95%)	
Stroke, *N* (%)					<0.05
Yes	612 (5.02%)	455 (3.73%)	382 (3.13%)	339 (2.78%)	
No	11568 (94.98%)	11744 (96.27%)	11826 (96.87%)	11863 (97.22%)	
Heart attack, *N* (%)					<0.05
Yes	750 (6.16%)	523 (4.29%)	462 (3.79%)	399 (3.27%)	
No	11424 (93.84%)	11673 (95.71%)	11739 (96.21%)	11801 (96.73%)	
CHD, *N* (%)					<0.05
Yes	726 (5.99%)	508 (4.18%)	423 (3.47%)	345 (2.83%)	
No	11386 (94.01%)	11653 (95.82%)	11763 (96.53%)	11828 (97.17%)	
Angina, *N* (%)					<0.05
Yes	454 (3.74%)	335 (2.75%)	301 (2.47%)	302 (2.48%)	
No	11691 (96.26%)	11844 (97.25%)	11872 (97.53%)	11882 (97.52%)	

Continuous variables were expressed as weighted means and standard deviations (SD). BMl, body mass index; NLR, neutrophil to Lymphocyte ratio; CHD, coronary heart disease; ASCVD, Atherosclerotic cardiovascular disease.

### 3.2 Association between ALI and ASCVD

**FIGURE 2:**
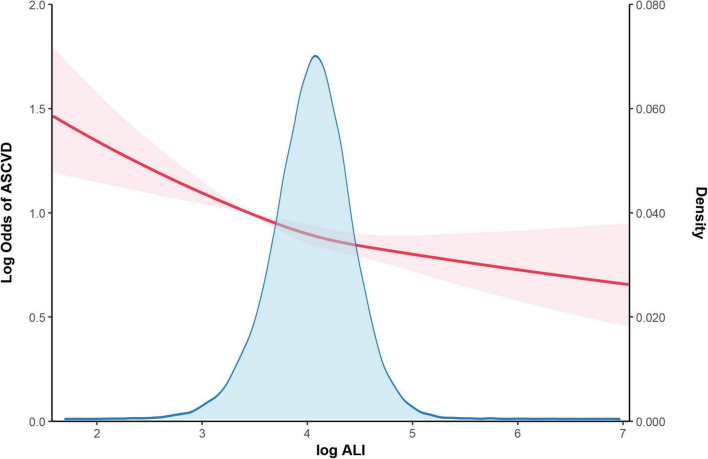
The association between log ALI and ASCVD. The solid red line represents the smooth curve fit between variables. Red bands represent the 95% of confidence interval from the fit. Controlled Attenuation Parameter; log ALI: advanced lung cancer inflammation (ALl) were converted to natural logarithm to correct for right skewness. We adjusted age, gender, race, poverty income ratio, smoking status, insurance, hypertension, diabetes and hyperlipidemia. ASCVD, atherosclerotic cardiovascular disease. Density: The Y-axis of the density histogram represents the relative frequency per unit interval, calculated as frequency divided by bin width. The area of each bar equals the density multiplied by the bin width, representing the frequency for that interval, and when normalized, the total area sums to 1.

The findings of the multivariate logistic regression analyses, as shown in Table 2, reveal a consistent and statistically significant inverse association between Ln-transformed ALI and the incidence of ASCVD across all three models examined. After adjusting for potential confounders, the data demonstrate that individuals with higher ALI values exhibit a lower prevalence of ASCVD. Specifically, each unit increase in higher Ln-transformed ALI is linked to lower probabilities of ASCVD prevalence (OR: 0.87, 95% CI: 0.82–0.92). Further analysis using quartiles of ALI corroborates this relationship, with adjusted odds ratios (OR) indicating progressively lower developing of ASCVD across increasing quartiles: 0.92 (95% CI: 0.84–1.00, *P* < 0.05) for the second quartile, 0.87 (95% CI: 0.79–0.95, *P* < 0.05) for the third quartile, and 0.82 (95% CI: 0.74–0.90, *P* < 0.05) for the highest quartile compared to the reference group in the lowest quartile. Furthermore, a generalized model incorporating smooth curve fitting provides additional evidence supporting the inverse relationship between Ln-transformed ALI and ASCVD, as depicted in Figure 2 and original one is in Supplementary Table 1B. These findings suggest that elevated Ln-transformed ALI levels are robustly associated with a reduced prevalence rate of developing ASCVD.

**TABLE 2:** Odds ratios (95%CI) of Ln-transformed ALI and atherosclerotic cardiovascular disease.

Exposure	Model 1 [OR (95%CI)]	*P*	Model 2 [OR (95%CI)]	*P*	Model 3 [OR (95%CI)]	*P*
Continuous ALI*	0.64 (0.60, 0.68)	<0.05	0.89 (0.83, 0.94)	<0.05	0.87 (0.82, 0.92)	<0.05
ALI* classification						
Q1 group	Reference		Reference		Reference	
Q2 group	0.70 (0.65, 0.76)	<0.05	0.93 (0.85, 1.01)	0.0769	0.92 (0.84, 1.00)	0.0492
Q3 group	0.60 (0.56, 0.65)	<0.05	0.89 (0.82, 0.97)	0.0113	0.87 (0.79, 0.95)	0.0027
Q4 group	0.54 (0.50, 0.59)	<0.05	0.85 (0.78, 0.94)	0.0009	0.82 (0.74, 0.90)	<0.05

Model 1: no covariates were adjusted. Model 2: age, gender, and race were adjusted. Model 3: age, gender, race, poverty income ratio, smoking status, insurance, hypertension, diabetes and hyperlipidemia were adjusted. ALI*: ALI were converted to natural logarithm to correct for right skewness, also as the Ln-transformed ALI for right skewness.

### 3.3 Subgroup analyses

To assess the overall population’s consistency with the relationship between ALI and ASCVD and look into impacts unique to subgroups, our study performed subgroup analyses and interaction tests across a range of factors, including poverty income ratio, race, gender, age, insurance status, hypertension, smoking status, diabetes, and hyperlipidemia (Table 3). Although relationships exhibited some variability, significant interactions were observed for gender (*P* < 0.05), whereas no statistically significant interactions were found for race, poverty income ratio, insurance status, smoking status, hypertension, hyperlipidemia, or diabetes (all *P* for interaction > 0.05). In conclusion, this study results reveal the relationship between Ln-transformed ALI and ASCVD is specifically dependent on gender (*P* < 0.05).

**TABLE 3:** Subgroup analysis of the association between Ln-transformed advanced lung cancer inflammation index and atherosclerotic cardiovascular disease.

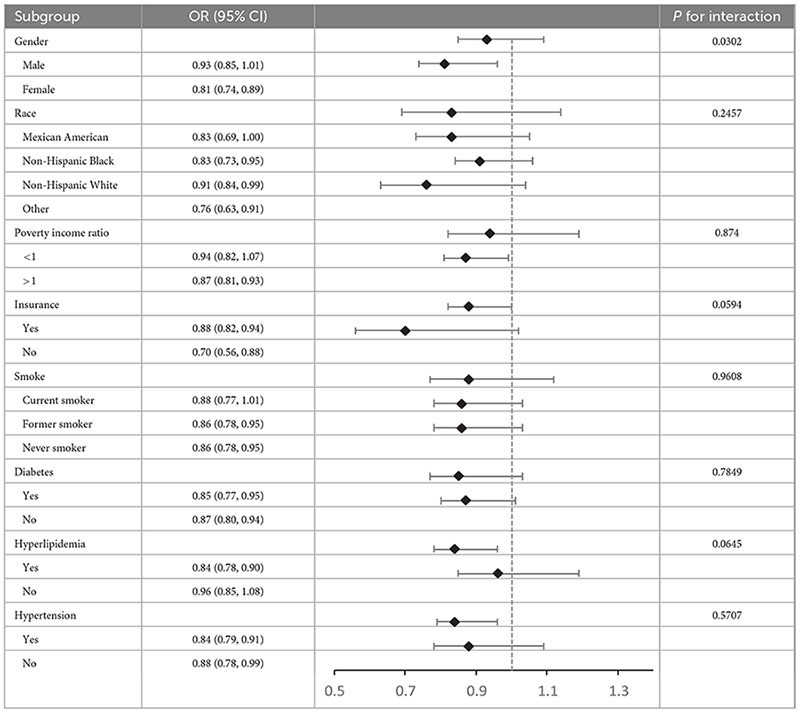

### 3.4 The discrimination powers of ALI, SII and PLR on ASCVD

**FIGURE 3:**
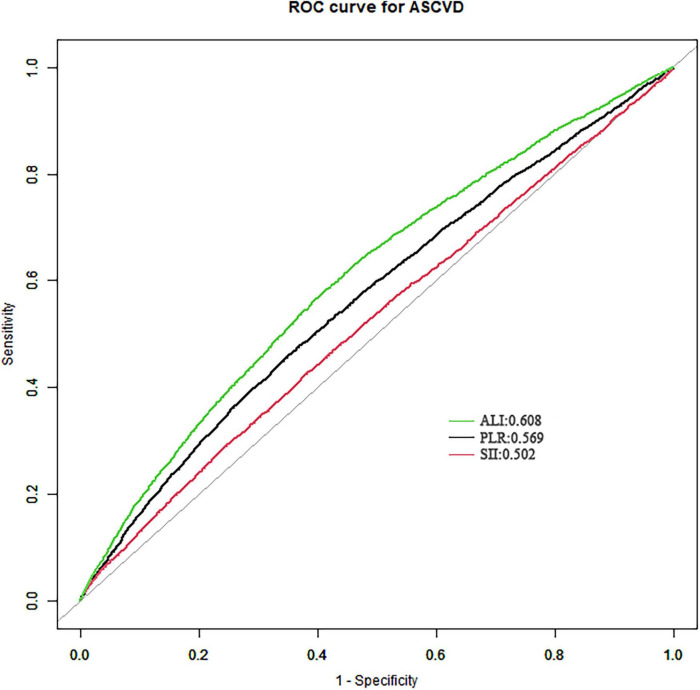
The receiver operating characteristics (ROC) analysis of ALI, SIl and PLR on ASCVD. AUC, the area under the curve; SII, systemic immune-inflammation index; PLR, platelet-to-lymphocyte ratio; ASCVD, atherosclerotic cardiovascular disease.

In the context of ROC curve analysis, an AUC closer to 1 signifies superior discriminative ability, whereas an AUC of 0.5 indicates a lack of discriminative power, equivalent to random chance. Among the markers analyzed, ALI exhibits the highest AUC at 0.608. Although this AUC value reflects limited discriminative capability, it surpasses those of SII (AUC = 0.502) and PLR (AUC = 0.569), demonstrating the statistically remarkable difference (*P* < 0.05) (Figure 3). Therefore, ALI, having the highest AUC among the three, emerges as the most effective predictor of ASCVD in comparison to SII and PLR.

## 4 Discussion

This study investigates the relationship between ASCVD and ALI in adult Americans via a cross-sectional approach. Our research sample included a total of 48,845 participants and presents evidence that higher ALI levels are linked to a lower probabilities of ASCVD prevalence in adults Americans. Subgroup analyses revealed a significant interaction effect based on gender. Furthermore, ALI emerged as a more accurate predictor of ASCVD surpassing both the SII and PLR. These findings indicate that ALI may be a valuable anthropometric marker for predicting the prevalence of ASCVD.

As far as we know, this research represents the first investigation into the association between ASCVD and ALI. In another research, Chen et al. found a noticeable correlation—which was unrelated to cancer mortality—between higher ALI levels and lower mortality from cardiovascular and all-cause diseases in individuals with Type 2 Diabetes Mellitus (11). According to the findings, keeping ALI within a particular range in clinical environments was essential for reducing patients’ mortality from cardiovascular disease as well as all causes in Type 2 Diabetes Mellitus patients. The analysis of Gender-stratified Restricted Cubic Spline suggested a linear negative relationship was observed between cardiovascular disease mortality and ALI in female Type 2 Diabetes Mellitus patients; in male patients, the result was consistent with the general population. Chen X et al. conducted a study involving 1,440 stroke patients and demonstrated that an elevated ALI was remarkably linked to a reduced risk of all-cause mortality, though no significant association was observed with cardiovascular disease mortality (8). There was no discernible interaction between the ALI and the stratification variables. Besides, Ren et al. (10) show the result that after included 277 participants a decreased ALI serves as an independent risk factor for both all-cause and cardiovascular mortality in patients undergoing peritoneal dialysis (10). These studies and ours have reached similar conclusions, namely a negative relationship between ALI and ASCVD. The ALI may function as a valuable prognostic marker for predicting clinical outcomes in patients receiving peritoneal dialysis.

Atherosclerotic cardiovascular disease is a complex, multifactorial condition marked by the buildup of lipids, inflammatory cells, as well as fibrous components in the arterial walls, resulting in the formation of plaques that may impede blood flow (20, 21). The progression of ASCVD is closely linked to systemic inflammation, which plays a pivotal role in both the initiation and advancement of atherosclerotic plaques (22, 23). Among these, the PLR and SII have garnered attention due to their simplicity and ease of calculation from routine blood tests (24, 25). ALI is an inflammation marker calculated based on BMI, albumin levels, and NLR. The higher ALI typically indicates better nutritional status and lower levels of inflammation. Also the high ALI value is generally associated with a more favorable prognosis, suggesting that the patient may respond better to treatment. In models 1, 2, and 3, the group with a higher ALI exhibited a stronger effect in reducing the incidence of ASCVD. So our results are consistent with the general conclusions regarding the role of the ALI. Subgroup analysis in this study revealed that the negative correlation was more pronounced in women, demonstrating that the relationship between higher ALI and lower ASCVD prevalence is particularly relevant when stratified by gender. In response to the observed interactions between ALI and ASCVD based on gender and age, our study provides a potentially biological explanation for these findings (26). Gender differences in the relationship between ALI and ASCVD may stem from distinct immune responses influenced by sex hormones. Estrogen in women has been shown to have anti-inflammatory properties, while testosterone in men may promote inflammation (27). These hormonal variations could contribute to the differential impact of ALI on cardiovascular health between men and women (28). However, interaction tests showed that this negative correlation between ALI and ASCVD was not influenced by race, PIR, insurance status, smoking, hypertension, hyperlipidemia, or diabetes (*P*-values for interaction all > 0.05), suggesting that these negative correlations are consistent across these demographic and clinical contexts. Research has demonstrated that an elevated PLR is linked to a heightened risk of adverse cardiovascular events, indicating its potential as a predictor of ASCVD (29, 30). Elevated SII levels have been correlated with poor cardiovascular outcomes, emphasizing its potential role in ASCVD risk stratification (31, 32). At the same time, our study demonstrated that ALI has a higher AUC value compared to SII and PLR, suggesting that ALI may offer better predictive performance for inflammation and nutritional status.

While the results are encouraging, it is important to recognize some of the study’s limitations. Firstly, the cross-sectional research design makes it more difficult to determine a causal relationship between ASCVD and ALI. While the association is strong, it is not possible to determine whether higher ALI scores directly protect against the development of ASCVD, or if other unmeasured factors are contributing to this relationship. Additionally, potential confounding factors, such as the presence of other chronic diseases or lifestyle factors not fully accounted for in the analysis, may have influenced the results. The study’s reliance on self-reported data for some variables, such as ASCVD diagnosis, also introduces the possibility of reporting bias. Furthermore, the results’ generalizability could be limited to the population of the United States. as the study sample was derived from American representative dataset. Different populations, particularly those with different ethnic or socioeconomic backgrounds, may exhibit different associations between ALI and ASCVD, necessitating further research in diverse studies. Further large-scale, more diverse researches are necessary to validate the findings of this research and to investigate the mechanistic pathways linking ALI and ASCVD. Additionally, further exploration of the underlying biological mechanisms linking ALI and cardiovascular health could provide deeper insights into how nutritional and inflammatory factors interact in the pathogenesis of atherosclerosis.

Although this study reveals a significant association between ALI and ASCVD through rigorous statistical methods, it is important to note that the cross-sectional design of the study inherently cannot establish causality. While we controlled for major confounding factors through multivariable adjustments, residual confounding, such as genetic susceptibility, environmental exposure differences, and dynamic changes in ALI, may still influence the interpretation of the results. Future research could employ the following strategies to validate causal hypotheses: (1) establish a prospective cohort to monitor the longitudinal changes in ALI and its temporal relationship with ASCVD events; (2) apply Mendelian randomization analysis, using genetic instruments related to ALI to avoid confounding bias; (3) combine multi-omics data to explore the molecular pathways through which ALI influences atherosclerosis (e.g., inflammation-metabolism axis regulation). These methods will help differentiate whether ALI serves as a risk marker or a causal mediator in the development of ASCVD.

## 5 Conclusion

This study presents evidence that higher ALI levels are associated with a lower odds of ASCVD prevalence in United States adults. ALI is a superior predictor of developing ASCVD compared to traditional inflammatory markers such as the PLR and SII. The study also underscores the potential of ALI to provide a more comprehensive assessment of cardiovascular risk by integrating measures of both inflammation and nutritional status.

## Data Availability

The original contributions presented in this study are included in this article/Supplementary material, further inquiries can be directed to the corresponding author.

## References

[1] Tsao C Aday A Almarzooq Z Anderson C Arora P Avery C Heart disease and stroke statistics-2023 update: A report from the American heart association. *Circulation.* (2023) 147:e93–621. 10.1161/CIR.0000000000001123 36695182 PMC12135016

[2] Stone N Robinson J Lichtenstein A Bairey Merz C Blum C Eckel R 2013 ACC/AHA guideline on the treatment of blood cholesterol to reduce atherosclerotic cardiovascular risk in adults: A report of the American college of cardiology/American heart association task force on practice guidelines. *J Am Coll Cardiol.* (2014) 63:2889–934. 10.1016/j.jacc.2013.11.002 24239923

[3] Michos, McEvoy J Blumenthal R. Lipid management for the prevention of atherosclerotic cardiovascular disease. *N Engl J Med.* (2019) 381:1557–67. 10.1056/NEJMra1806939 31618541

[4] Birkenfeld A Franks P Mohan V. Precision medicine in people at risk for diabetes and atherosclerotic cardiovascular disease: A fresh perspective on prevention. *Circulation.* (2024) 150:1910–2. 10.1161/CIRCULATIONAHA.124.070463 39652647

[5] Lidgard B Zelnickv L Anderson A Feldman H Go A He J Cardiac biomarkers and risk of atherosclerotic cardiovascular disease in patients with CKD. *Kidney360.* (2022) 3:859–71. 10.34067/KID.0006222021 36128494 PMC9438429

[6] Kvist-Hansen A Kaiser H Krakauer M Gørtz P Wang X Becker C Neutrophil-to-lymphocyte ratio and the systemic immune-inflammation index as potential biomarkers of effective treatment and subclinical atherosclerotic cardiovascular disease in patients with psoriasis. *J Eur Acad Dermatol Venereol.* (2023) 37:e586–9. 10.1111/jdv.18860 36645810

[7] Gupta L Thomas J Ravichandran R Singh M Nag A Panjiyar B. Inflammation in cardiovascular disease: A comprehensive review of biomarkers and therapeutic targets. *Cureus.* (2023) 15:e45483. 10.7759/cureus.45483 37859889 PMC10584272

[8] Chen X Hong C Guo Z Huang H Ye L. Association between advanced lung cancer inflammation index and all-cause and cardiovascular mortality among stroke patients: NHANES, 1999-2018. *Front Public Health.* (2024) 12:1370322. 10.3389/fpubh.2024.1370322 38699426 PMC11063327

[9] Tu J Wu B Xiu J Deng J Lin S Lu J Advanced lung cancer inflammation index is associated with long-term cardiovascular death in hypertensive patients: National health and nutrition examination study, 1999-2018. *Front Physiol.* (2023) 14:1074672. 10.3389/fphys.2023.1074672 37206362 PMC10189044

[10] Ren Z Wu J Wu S Zhang M Shen S. The advanced lung cancer inflammation index is associated with mortality in peritoneal dialysis patients. *BMC Nephrol.* (2024) 25:208. 10.1186/s12882-024-03645-4 38918730 PMC11201339

[11] Chen Y Guan M Wang R Wang X. Relationship between advanced lung cancer inflammation index and long-term all-cause, cardiovascular, and cancer mortality among type 2 diabetes mellitus patients: NHANES, 1999-2018. *Front Endocrinol (Lausanne).* (2023) 14:1298345. 10.3389/fendo.2023.1298345 38111710 PMC10726345

[12] Wang X Wei C Fan W Sun L Zhang Y Sun Q Advanced lung cancer inflammation index for predicting prognostic risk for patients with acute coronary syndrome undergoing percutaneous coronary intervention. *J Inflamm Res.* (2023) 16:3631–41. 10.2147/JIR.S421021 37641701 PMC10460579

[13] Ma Z Wu S Guo Y Ouyang S Wang N. Association of advanced lung cancer inflammation index with all-cause and cardiovascular mortality in US patients with rheumatoid arthritis. *Front Nutr.* (2024) 11:1397326. 10.3389/fnut.2024.1397326 38873560 PMC11169605

[14] Chen X Li A Ma Q. Neutrophil-lymphocyte ratio and systemic immune-inflammation index as predictors of cardiovascular risk and mortality in prediabetes and diabetes: A population-based study. *Inflammopharmacology.* (2024) 32:3213–27. 10.1007/s10787-024-01559-z 39167310

[15] Gong M Sasmita B Zhu Y Chen S Wang Y Xiang Z Prognostic value of the advanced lung cancer inflammation index ratio in patients with acute myocardial infarction complicated by cardiogenic shock: A cohort study. *Rev Cardiovasc Med.* (2024) 25:267. 10.31083/j.rcm2507267 39139443 PMC11317351

[16] Liang Z Feng Y Shivappa N Hebert J Xu X. Dietary inflammatory index and mortality from all causes, cardiovascular disease, and cancer: A prospective study. *Cancers (Basel).* (2022) 14:4609. 10.3390/cancers14194609 36230532 PMC9564054

[17] Curtin L Mohadjer L Dohrmann S Montaquila J Kruszan-Moran D Mirel L The national health and nutrition examination survey: Sample design, 1999-2006. *Vital Health Stat.* (2012) 155:1–39. 22788053

[18] US Department of Health & Human Services. *Office of extramural research.* Washington, DC: US Department of Health & Human Services (2021).

[19] Perperoglou A Sauerbrei W Abrahamowicz M Schmid M. A review of spline function procedures in R. *BMC Med Res Methodol.* (2019) 19:46. 10.1186/s12874-019-0666-3 30841848 PMC6402144

[20] Libby P Ridker P Hansson G Leducq Transatlantic Network on Atherothrombosis. Inflammation in atherosclerosis: From pathophysiology to practice. *J Am Coll Cardiol.* (2009) 54:2129–38. 10.1016/j.jacc.2009.09.009 19942084 PMC2834169

[21] Pahwa R Jialal I. *Atherosclerosis: StatPearls.* Treasure Island, FL: StatPearls Publishing (2024).

[22] Hansson GK. Inflammation and atherosclerosis: The end of a controversy. *Circulation.* (2017) 136:1875–7. 10.1161/CIRCULATIONAHA.117.030484 28916641

[23] Ridker P Everett B Thuren T MacFadyen J Chang W Ballantyne C Antiinflammatory therapy with canakinumab for atherosclerotic disease. *N Engl J Med.* (2017) 377:1119–31. 10.1056/NEJMoa1707914 28845751

[24] Yüksel M Yildiz A Oylumlu M Akyüz A Aydin M Kaya H The association between platelet/lymphocyte ratio and coronary artery disease severity. *Anatol J Cardiol.* (2015) 15:640. 10.5152/akd.2014.5565 25550173 PMC5336865

[25] Hu B Yang X Xu Y Sun Y Sun C Guo W Systemic immune-inflammation index predicts prognosis of patients after curative resection for hepatocellular carcinoma. *Clin Cancer Res.* (2014) 20:6212–22. 10.1158/1078-0432.CCR-14-0442 25271081

[26] Zhang H Jiao L Yang S Li H Jiang X Feng J Brain-computer interfaces: The innovative key to unlocking neurological conditions. *Int J Surg.* (2024) 110:5745–62. 10.1097/JS9.0000000000002022 39166947 PMC11392146

[27] Zhang H Chen Y Jiang X Gu Q Yao J Wang X Unveiling the landscape of cytokine research in glioma immunotherapy: A scientometrics analysis. *Front Pharmacol.* (2024) 14:1333124. 10.3389/fphar.2023.1333124 38259287 PMC10800575

[28] Zhang H Jiang X Li A Wang X. Causal associations between gut microbiota and cerebrovascular diseases. *World Neurosurg.* (2024) 183:e587–97. 10.1016/j.wneu.2023.12.150 38191059

[29] Zahorec R. Ratio of neutrophil to lymphocyte counts–rapid and simple parameter of systemic inflammation and stress in critically ill. *Bratisl Lek Listy.* (2001) 102:5–14. 11723675

[30] Kurtul A Murat SN Yarlioglues M Duran M Ergun G Acikgoz SK Association of platelet-to-lymphocyte ratio with severity and complexity of coronary artery disease in patients with acute coronary syndromes. *Am J Cardiol.* (2014) 114:972–8. 10.1016/j.amjcard.2014.07.005 25118117

[31] Gibson P Croal B Cuthbertson B Small G Ifezulike A Gibson G Preoperative neutrophil-lymphocyte ratio and outcome from coronary artery bypass grafting. *Am Heart J.* (2007) 154:995–1002. 10.1016/j.ahj.2007.06.043 17967611

[32] Ridker P. From C-reactive protein to interleukin-6 to interleukin-1: Moving upstream to identify novel targets for atheroprotection. *Circ Res.* (2016) 118:145–56. 10.1161/CIRCRESAHA.115.306656 26837745 PMC4793711

