# Supplementary material for: Association between advanced lung cancer inflammation index and atherosclerosis cardiovascular disease in U.S. adults: a population-based investigation
Source: Front Nutr. 2025 Jun 4;12:1503844. doi: 10.3389/fnut.2025.1503844 (PMC12173879; doi:10.3389/fnut.2025.1503844)

# Supplementary Table 1A. Disease diagnosis from NHANES database.

| **Disease** | **Section** | **English Text** | **Value Description for Diagnosis Yes** |
| --- | --- | --- | --- |
| angina | Questionnaire Data | Has a doctor or other health professional ever told {you/SP} that {you/s/he} . . .had angina (an-gi-na), also called angina pectoris? | Yes |
| heart attack | Questionnaire Data | Has a doctor or other health professional ever told {you/SP} that {you/s/he} . . .had a heart attack (also called myocardial infarction (my-o-car-dee-al in-fark-shun))? | Yes |
|  | Questionnaire Data | Has a doctor or other health professional ever told {you/SP} that {you/he/she} had …? | a heart attack? |
| stroke | Questionnaire Data | Has a doctor or other health professional ever told {you/SP} that {you/s/he} . . .had a stroke? | Yes |
|  | Questionnaire Data | Has a doctor or other health professional ever told {you/SP}that {you/he/she} had . . .? | a stroke? |
| coronary heart disease | Questionnaire Data | Has a doctor or other health professional ever told {you/SP} that {you/s/he} . . .had coronary (kor-o-nare-ee) heart disease? | Yes |
| diabetes | Questionnaire Data | The next questions are about specific medical conditions. {Other than during pregnancy, {have you/has SP}/ {Have you/Has SP}} ever been told by a doctor or health professional that {you have/{he/she/SP} has} diabetes or sugar diabetes? | Yes |
|  | Laboratory Data | Glycohemoglobin | > 6.5% |
|  | Laboratory Data | Fasting glucose | ≥7.0 mmol/L |
|  | Laboratory Data | random blood glucose | ≥11.1 mmol/L |
|  | Laboratory Data | Two Hour Glucose (OGTT) | ≥11.1 mmol/L |
|  | Questionnaire Data | Use of diabetes medication or insulin, including biguanides (metformin), sulfonylureas, insulin, thiazolidinediones, dipeptidyl peptidase 4 inhibitors, glucose like peptide-1 receptor agonists, sodium-glucose co-transporter-2 inhibitors, alpha-glucosidase inhibitors, meglitinides, amylin analogs, other antidiabetic agents. |  |
| hyperlipidemia | Questionnaire Data |  |  |
|  | Laboratory Data | TG | ≥150 mg/dL |
|  | Laboratory Data | TC | ≥200 mg/dL |
|  | Laboratory Data | LDL-C | ≥130 mg/dL |
|  | Laboratory Data | HDL-C | ≤40mg/dL (male), 50mg/dL (female) |
|  | Questionnaire Data | Use of cholesterol-lowering drugs, including statins, including statins, ezetimibe, niacin and its derivatives, phenoxyaryl acids and their derivatives. |  |
| hypertension | Questionnaire Data | {Were you/Was SP} told on 2 or more different visits that {you/s/he} had hypertension, also called high blood pressure? | Yes |
|  | Questionnaire Data | {Have you/Has SP} ever been told by a doctor or other health professional that {you/s/he} had hypertension, also called high blood pressure? | Yes |
|  | Examination Data | Average SBP, average DBP (at least 3 times) | Average SBP≥140 mmHg, average DBP≥90 mmHg |
|  | Questionnaire Data | Use of antihypertensive medication, including angiotensin converting enzyme inhibitors, angiotensin receptor blockers, diuretics, calcium channel blockers, beta blockers, alpha blockers, centrally acting agents, direct vasodilators, aldosterone receptor antagonists, renin inhibitors, other antihypertensive agents. |  |

222

# Supplementary Table 1B. Smooth curve fitting plot between ASCVD and ALI without transformation.


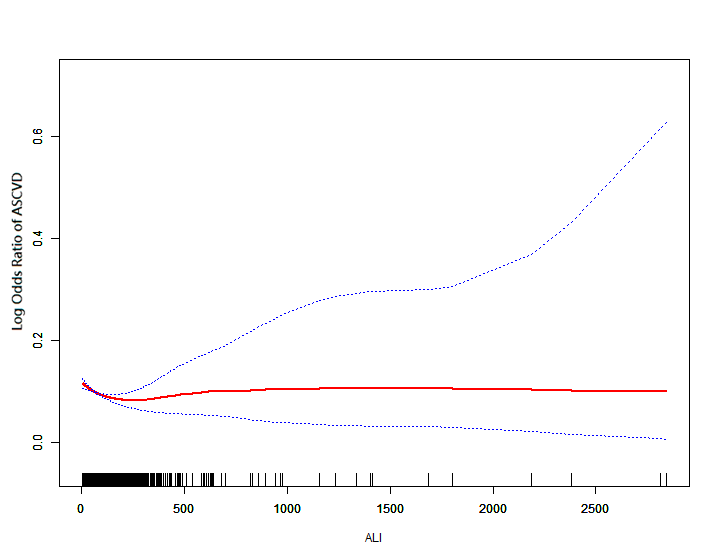

Supplement: Supplementary file 1 [file Table_1.docx]
